# The role of glial cells in neuralgia: a bibliometric exploration

**DOI:** 10.3389/fneur.2025.1496526

**Published:** 2025-02-07

**Authors:** Ting He, DongDong Wang, Linman Wu, Liuyin Jin

**Affiliations:** ^1^Shaoxing Seventh People’s Hospital, Shaoxing, China; ^2^Peking University Medical Zibo Hospital, Zibo, China; ^3^Nanchong Mental Health Center of Sichuan Province, Nanchong, China; ^4^The Second People's Hospital of Lishui, Lishui, China

**Keywords:** neuropathic pain, neuralgia, glia, research hotspots, bibliometrics, data visualization

## Abstract

Neuropathic pain is a chronic pathological pain caused by nervous system damage, characterized by complex mechanisms and limited treatment efficacy. Glial cells play a pivotal role in the initiation and maintenance of neuropathic pain. This study employs bibliometric analysis to explore trends and emerging hotspots in research on the relationship between glial cells and neuropathic pain. Based on literature data from the Web of Science Core Collection spanning 2003 to 2022, the study identifies key contributors in the field, including leading countries such as China, the United States, and Japan, as well as influential institutions and journals, such as the University of California system and Pain. Keyword analysis highlights research hotspots focusing on glial cell activation, spinal cord injury, satellite glial cells, oxidative stress, and neuroinflammation. The findings suggest that these themes may shape future directions in the field. This study provides researchers with a comprehensive overview of trends and hotspot analysis, offering valuable insights for further investigation into the role of glial cells in neuropathic pain.

## 1 Introduction

Neuropathic pain encompasses a range of conditions caused by lesions or diseases in the parts of the nervous system that transmit somatosensory information. These conditions include various peripheral nervous system disorders such as postherpetic neuralgia, painful neuropathy, trigeminal neuralgia, post-amputation pain, and other neuropathies. The diseases leading to neuropathic pain vary widely in anatomical location and etiology. Despite this diversity, neuropathic pain shares common clinical features, including pain in areas of partial or complete sensory loss and different types of provoked pain (1–4). Prominent symptoms include hyperalgesia and allodynia (5), and the pain is often described as burning, electrical, or stabbing. Unlike nociceptive pain, neuropathic pain is frequently associated with “positive” symptoms such as tingling and tightness and “negative” symptoms such as numbness, sensory loss, or a sensation of “falling asleep” (6). Chronic neuropathic pain can severely reduce quality of life, impairing sleep, work, and daily activities, and it is often associated with depressive moods. Persistent pain weakens emotional resilience, triggers frustration, and exacerbates mental health issues like low mood and anxiety, which in turn intensify the perception of pain (7). Despite advances in pain management, effective treatment of neuropathic pain remains limited due to its multifaceted etiology.

Recent research has highlighted the critical role of glial cells in the development and maintenance of neuropathic pain. Traditionally considered supportive elements in the nervous system, glial cells are increasingly recognized for their role in pain regulation and synaptic plasticity (8, 9). Microglia, the resident immune cells of the central nervous system, are involved in neuroinflammation following nerve damage (10). Astrocytes, essential for neurohomeostasis and synaptic function, undergo reactive changes during nerve injury, contributing to neuroinflammation and hypersensitivity (11–15). Neuron-glial cell interactions involve the release of pro-inflammatory cytokines, chemokines, and other signaling molecules that modulate the pain experience (16, 17).

Bibliometric analysis, a method for quantitatively and qualitatively evaluating literature, provides a comprehensive understanding of research trends, relationships, and clusters. It assesses the contributions and impact of authors, countries, institutions, and journals while identifying emerging trends and research frontiers (18). This study applies bibliometric methods to analyze neuropathic pain research involving glial cells. By examining publication trends, co-authorship networks, and citation patterns, it identifies key contributors, influential studies, and active research areas, offering an overview of the field and highlighting potential knowledge gaps.

## 2 Data and methods

### 2.1 Literature search and screening

We conducted a bibliometric analysis using the Web of Science database, which offers significant advantages for bibliometric research compared to other databases. First, Web of Science covers a broader range of academic disciplines. Second, it has a longer time span and includes various types of data. Most importantly, Web of Science provides powerful citation analysis tools, which are essential for detailed citation, trend, and impact analysis. Our literature search process was as follows: 1. Defining the search strategy: We used topic search (TS) = (“neuralgia” OR “neuropathic pain” OR “neuralgic pain” OR “nerv* pain”) AND TS = (“glia” OR “spongiocyte” OR “colloidcell” OR “neuroglia* cell*” OR “schwann cells” OR “glioma cells” OR “gail cells” OR “microglia cell” OR “gilacells” OR “gliocyte”). 2. Advanced Search: We utilized the advanced search functionality of the Web of Science database to perform our search. 3. Inclusion and Exclusion Criteria: Inclusion Criteria: (1) Articles published between 2003 and 2022; (2) Articles written in English; (3) Articles classified as reviews or research papers. Exclusion Criteria: (1) Studies unrelated to glial cells or neuropathic pain; (2) Non-academic articles, such as conference abstracts, book reviews, and editorials; (3) Duplicate publications.

### 2.2 Bibliometrics and visual analysis

In this study, we utilized various bibliometric tools for data extraction and analysis, including the bibliometrix R package, CiteSpace, and VOSviewer. The bibliometrix package^1^ is a comprehensive R-based tool designed for bibliometric analysis, offering a wide range of functions to process and analyze large-scale document datasets. It enables researchers to perform various analyses such as co-authorship network analysis, keyword analysis, citation analysis, and patterns of author collaboration, helping to explore research trends and hotspots in neuropathic pain and glial cell studies. Using bibliometrix, we calculated metrics such as publication count, author contributions, and citation impact. Additionally, it allowed us to generate time trend plots and author collaboration networks to gain deeper insights into the field’s development (19). Collaboration frequency was calculated by analyzing author affiliation data extracted from publications, including countries or institutions. The process involved extracting this information from the author metadata, defining collaboration as any publication authored by researchers from multiple countries or institutions, and calculating collaboration frequencies by counting co-authored publications at national or institutional levels. For example, in a single publication involving three authors from countries A, B, and C, the collaborations A-B, A-C, and B-C were each counted once.

CiteSpace is another bibliometric analysis tool designed for analyzing and visualizing scientific literature. It identifies trends, key topics, author collaboration networks, and knowledge structures by examining citation relationships and co-occurrence patterns. CiteSpace’s capabilities include co-occurrence analysis, citation relationship analysis, time trend analysis, co-authorship network visualization, and uncovering research frontiers. These features provide valuable insights into the interdisciplinary and cutting-edge aspects of neuropathic pain and glial cell research (20, 21).

VOSviewer (Visualization of Similarities Viewer) is a free bibliometric and visualization tool used to analyze and display relationships among keywords, topics, and authors in scientific datasets. Its features include keyword co-occurrence analysis, clustering analysis, mapping visualization, co-authorship network analysis, and time trend analysis. The interactive capabilities of VOSviewer allow researchers to identify research hotspots, key publications, and trends in knowledge evolution within neuropathic pain and glial cell studies (22). Additionally, we used the ggplot2 package to enhance the visualization of our analysis results, producing clear and detailed graphical representations of research trends and hotspots (23, 24).

For data extraction and analysis, we first exported bibliometric data from the Web of Science database, including fields such as article titles, authors, publication years, journal names, keywords, abstracts, and citation counts, in .txt format. The bibliometrix package was then used to process this data, calculate key metrics such as publication counts, author contributions, and citation impact, and generate visualizations like time trend graphs and author collaboration networks. Using VOSviewer, we conducted co-citation analysis (e.g., author and document co-citation) and keyword co-occurrence analysis to reveal interrelationships among documents and identify research frontiers. Cluster analysis was performed with the focus on document co-citation or keyword co-occurrence, using suitable clustering algorithms and thresholds to create cluster maps that highlighted research hotspots and key themes. CiteSpace was employed to analyze temporal trends in research themes, identify evolutionary changes, and generate burst detection analysis to highlight emerging areas of interest. By identifying high-frequency keywords, frontier research, and influential citations within specific time periods, CiteSpace helped us understand the dynamic evolution of the field and predict future research directions. Finally, ggplot2 was utilized to refine and enhance the visualization of our results, offering clear and impactful displays of the identified trends and hotspots. This comprehensive approach enabled us to systematically analyze and interpret bibliometric data, uncovering significant trends, collaborations, and research priorities in the field of neuropathic pain and glial cell studies.

## 3 Results

### 3.1 Search results

**Figure 1:**
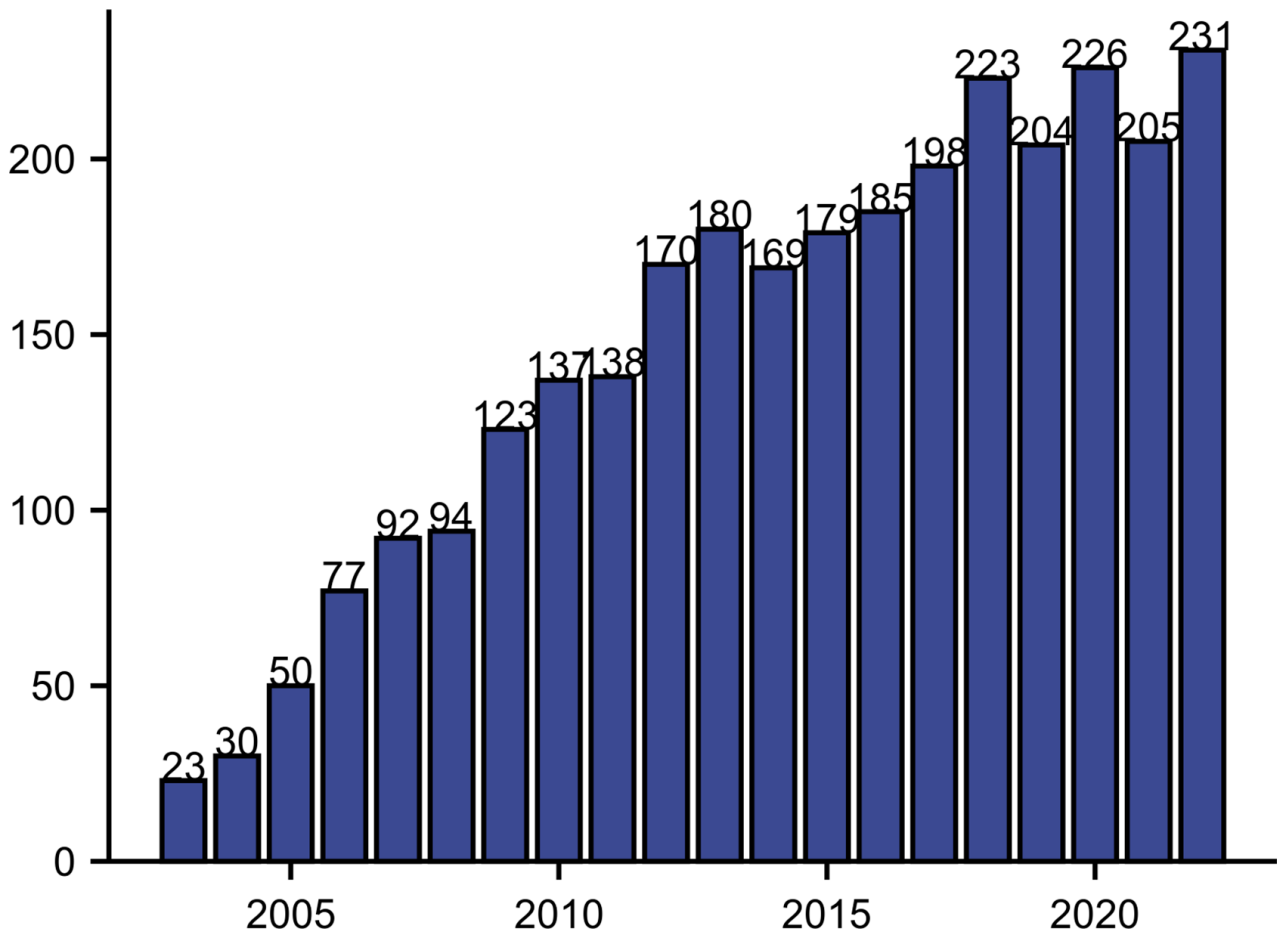
Annual publication count on neuropathic pain and glial cells from 2000 to 2022.

We found a total of 3,293 articles, excluding articles in languages other than English and articles with non-review or monograph types, and the search results showed 2,956 articles in the Web of Science database from 2003 to 2022. After importing the data into Bibliometrix to exclude the literature that did not meet the requirements, 2,934 articles were obtained, including 2,437 articles in the monograph category and 497 articles in the review category. Since 2009, the number of articles on neuralgia and glial cells has exceeded 100, and since then the number of neuralgia and glial cell related studies has remained at more than 100. By 2022, the number of research articles related to neuralgia and glial cells reached a maximum of 231, as shown in Figure 1.

### 3.2 Literature distribution characteristics

**Figure 2:**
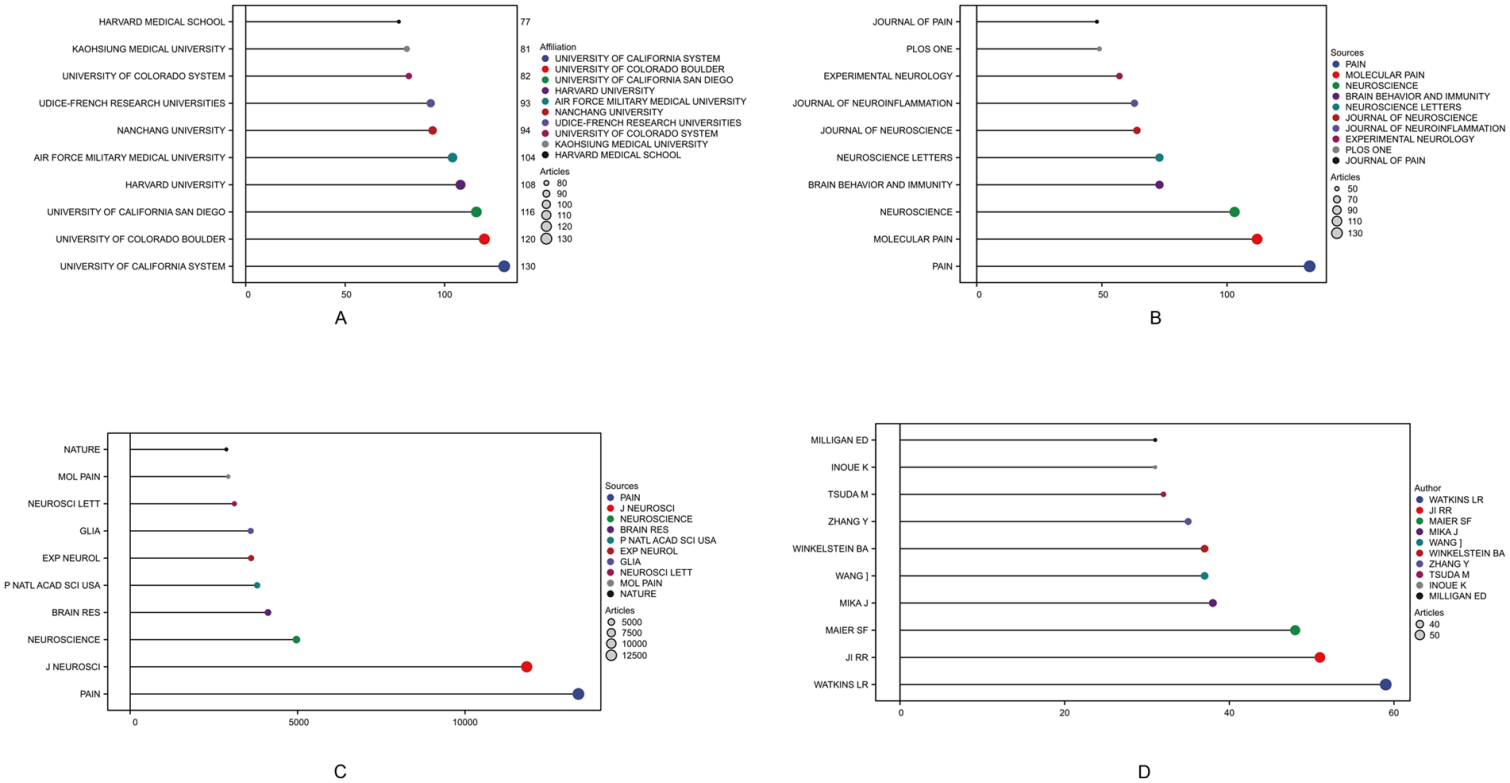
Top 10 institutions, journals by publication volume, journals by citation count, and authors by publication volume in the field of neuropathic pain and glial cells research from 2003 to 2022. The different colored circles represent distinct institutions, journals, or authors, with larger circles indicating higher counts. **(A)** Displays the top-ranking institutions by publication volume, reflecting their contributions and influence in this research field. **(B)** Shows the distribution of journals with the highest publication volume, highlighting the core journals with significant academic influence in this field. **(C)** Illustrates journal citation counts, indicating the relative impact of different journals in terms of literature dissemination and academic influence. **(D)** Depicts contributions at the author level, showcasing the top 10 authors by publication volume. The different colored circles represent distinct institutions, journals, or authors, with larger circles indicating higher counts.

Among the total 56 countries and regions, China ranked first in the number of articles published (*N* = 803, 27.37%), followed by the United States (*N* = 750, 25.56%) and Japan (*N* = 270, 9.20%). Among them, the United States has the highest rate of multiple country publications (MCP), indicating that it has closer cooperation between countries (Table 1). In 1981, the top three institutions published were THE UNIVERSITY OF CALIFORNIA SYSTEM (130) ranked first, followed by THE UNIVERSITY OF COLORADO BOULDER (120) and THE UNIVERSITY OF CALIFORNIA SAN DIEGO (116) as shown in Figure 2A. A total of 612 journals were published, and the top 10 journals published a total of 775 papers, of which the journal “Pain” (*N* = 133) had the largest number of publications (Figure 2B). The most cited journal in the total number of journals published was Pain, which totaled 13,452 times, as shown in Figure 2C. A total of 10,339 authors were included, and the top five authors were WATKINS LR (*N* = 59), JI RR (*N* = 51), MAIER SF (*N* = 48), MIKA J (*N* = 38), WANG J (*N* = 37), with a total of 233 articles (7.94%) (Figure 2D).

**Table 1:** Top 10 countries or regions in the field of neuralgia and glial cell research from 2003 to 2022.

Number	Country	Articles	SCP	MCP	Freq
1	China	803	702	101	0.274
2	USA	750	571	179	0.256
3	Japan	270	229	41	0.092
4	Italy	137	101	36	0.047
5	Korea	99	82	17	0.034
6	Germany	83	54	29	0.028
7	Brazil	80	57	23	0.027
8	Canada	79	60	19	0.027
9	Australia	74	47	27	0.025
10	United Kingdom	74	54	20	0.025

### 3.3 Literature co-citation analysis

In the literature co-citation analysis, the most cited in the world is “The neuropathic pain triad: neurons, immune cells and glia” published by SCHOLZ J et al. in NAT NEUROSCIi, with a total of 1,275 citations. In second place was MILLIGAN ED et al.’s “Pathological and protective roles of glia in chronic pain” published in MILLIGAN ED, with a total of 1,030 citations. IN THIRD PLACE WAS “MAP KINASE AND PAIN” PUBLISHED BY JI RR ET AL. IN BRAIN RES REV, WITH A TOTAL OF 768 CITATIONS. The rest are shown in Table 2. The most cited local is “The neuropathic pain triad: neurons, immune cells and glia” published in NAT NEUROSCI et al., with a total of 394 citations. In second place was “Pathological and protective roles of glia in chronic pain” by MILLIGAN ED et al., published in NAT REV NEUROSCI, with a total of 368 citations. In third place is Table 3 of “Inhibition of microglial activation attenuates the development but not existing hypersensitivity in a rat model of neuropathy” published in J PHARMACOL EXP THER.

**Table 2:** Top 10 cited articles in the field of neuralgia and glial cell research from 2003 to 2022.

Number	First author	Article name	Periodical	Year	Total citations
1	Scholz J	The neuropathic pain triad: neurons, immune cells and glia	NAT NEUROSCI	2007	394
2	MillIgan ED	Pathological and protective roles of glia in chronic pain	NAT REV NEUROSCI	2009	368
3	Raghaendav	Inhibition of microglial activation attenuates the development but not existing hypersensitivity in a rat model of neuropathy	J PHARMACOL EXP THER	2003	324
4	JI RR	Glia and pain: is chronic pain a gliopathy?	PAIN	2013	267
5	Tsuda M	Neuropathic pain and spinal microglia: a big problem from molecules in “small” glia	TRENDS NEUROSCI	2005	253
6	Ledeboer A	Minocycline attenuates mechanical allodynia and proinflammatory cytokine expression in rat models of pain facilitation	PAIN	2005	253
7	Jin SX	p38 mitogen-activated protein kinase is activated after a spinal nerve ligation in spinal cord microglia and dorsal root ganglion neurons and contributes to the generation of neuropathic pain	J NEUROSCI	2003	252
8	Watkins LR	Glia: a novel drug discovery target for clinical pain	TRENDS NEUROSCI	2003	645
9	Milligan ED	Spinal glia and proinflammatory cytokines mediate mirror-image neuropathic pain in rats	J NEUROSCI	2003	244
10	Zhuang ZY	ERK is sequentially activated in neurons, microglia, and astrocytes by spinal nerve ligation and contributes to mechanical allodynia in this neuropathic pain model	PAIN	2005	238

**Table 3:** Top ten cited Local articles in the field of neuralgia and glial cell research from 2003 to 2022.

Number	First author	Article name	Periodical	Year	Total citations
1	Scholz J	The neuropathic pain triad: neurons, immune cells and glia	NAT NEUROSCI	2007	1,275
2	Milligan ED	Pathological and protective roles of glia in chronic pain	NEUROSCI	2009	1,030
3	JI RR	MAP kinase and pain	BRAIN RES REV	2009	768
4	JI RR	Glia and pain: is chronic pain a gliopathy?	PAIN	2013	740
5	Jin SX	p38 mitogen-activated protein kinase is activated after a spinal nerve ligation in spinal cord microglia and dorsal root ganglion neurons and contributes to the generation of neuropathic pain	J NEUROSCI	2003	703
6	JI RR	Pain regulation by non-neuronal cells and inflammation	J NEUROSCIE	2016	679
7	Raghaendav	Inhibition of microglial activation attenuates the development but not existing hypersensitivity in a rat model of neuropathy	J PHARMACOL EXP THER	2003	650
8	Tsuda M	Neuropathic pain and spinal microglia: a big problem from molecules in “small” glia	TRENDS NEUROSCI	2005	644
9	Zhuang ZY	ERK is sequentially activated in neurons, microglia, and astrocytes by spinal nerve ligation and contributes to mechanical allodynia in this neuropathic pain model	PAIN	2005	619
10	JI RR	Emerging targets in neuroinflammation-driven chronic pain	REV DRUG DISCOV	2014	606

### 3.4 Analysis of national and institutional cooperation

**Figure 3:**
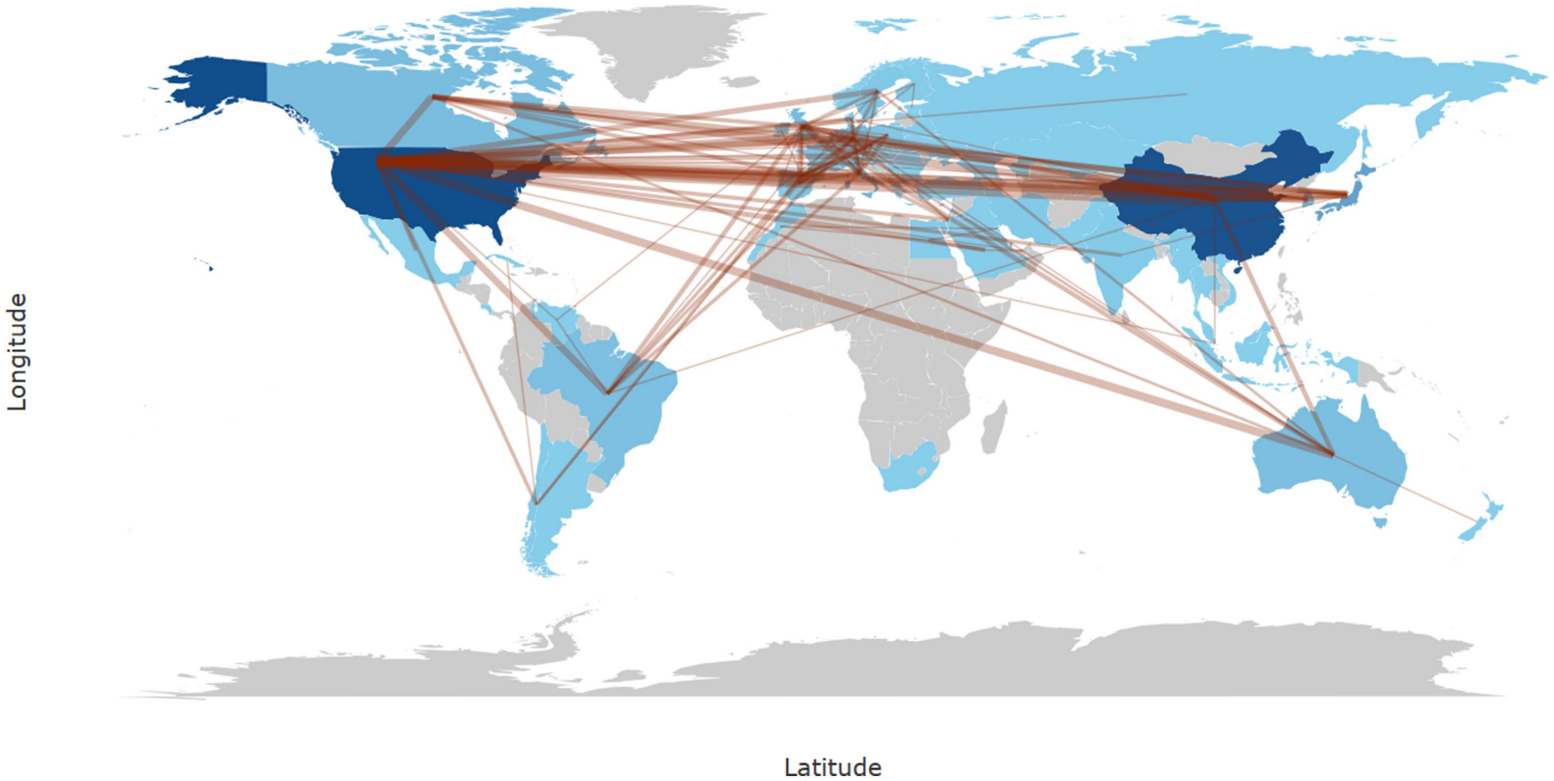
World map of international research collaborations in the field of neuropathic pain and glial cells from 2003 to 2022. The intensity of blue shading indicates the number of publications, with darker shades representing higher publication counts in that region. The thickness of brown lines represents the strength of collaboration based on frequency, with thicker lines indicating stronger collaboration between regions.

In Bibliometrix, the calculation of collaboration frequency is based on the country information of co-authors in the literature, counting the number of co-authored publications for each pair of countries. For instance, if a publication includes authors from both China and the United States, it is recorded as one instance of collaboration between these two countries. In cases involving multiple countries, the collaborations are split into pairwise records and cumulatively counted. In our study, the most frequent collaboration was between China and the United States (frequency = 129), followed by collaborations between the United States and Japan (frequency = 36), the United States and Italy (frequency = 33), the United States and Australia (frequency = 31), and the United States and South Korea (frequency = 28) (Figure 3).

### 3.5 Keyword analysis and collinearization

**Figure 4:**
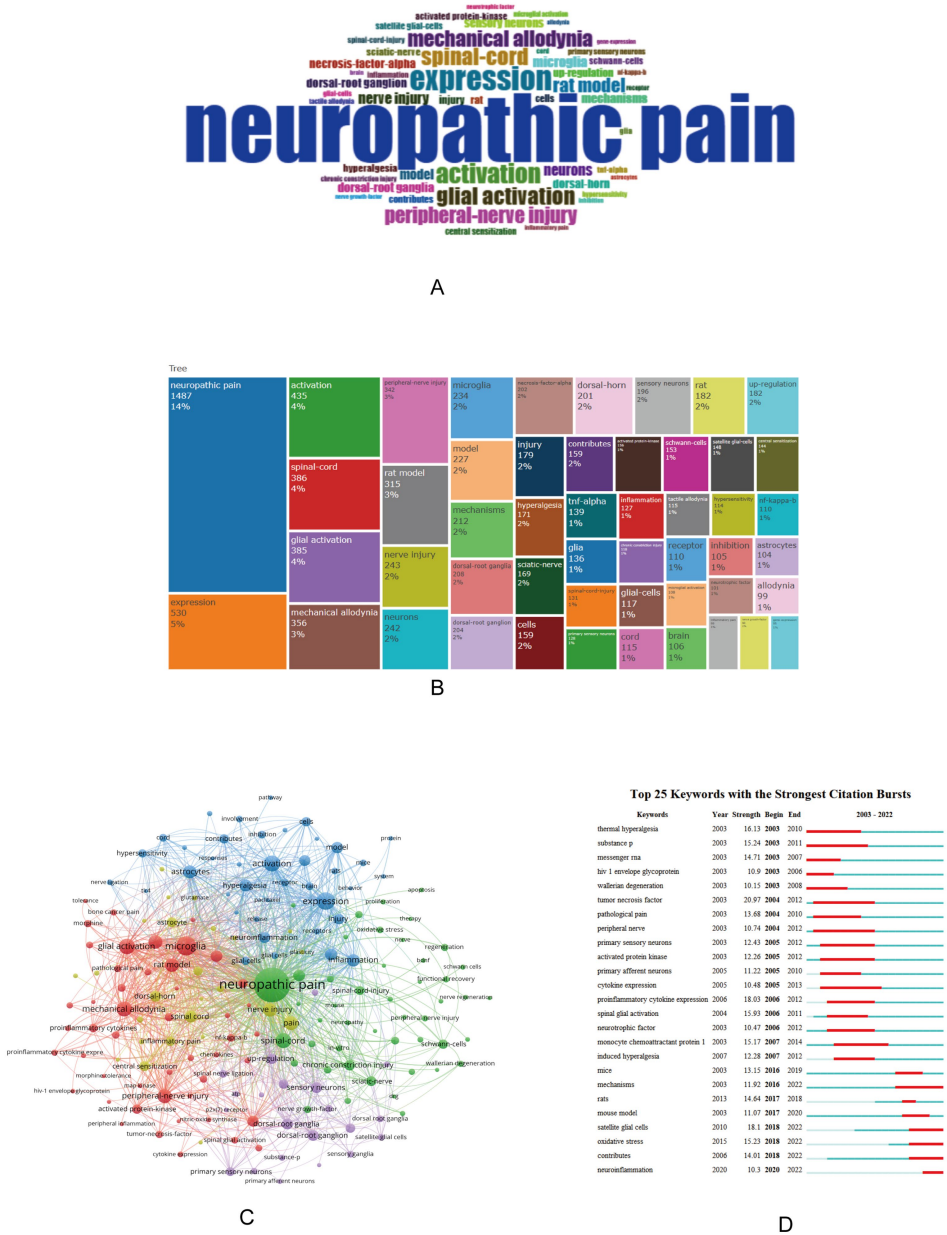
Word cloud **(A)**, top 50 keywords tree map **(B)**, keyword co-occurrence clustering map **(C)**, and keyword burst detection map **(D)** in the field of neuropathic pain and glial cells research from 2003 to 2022. **(A,B)** Results of keyword analysis using Bibliometrix. **(A)** is a word cloud, where the size of each word reflects its frequency, with larger areas indicating higher occurrence. **(B)** is a tree map illustrating the top 50 keywords, highlighting core keywords and research hotspots in neuropathic pain and glial cells research. **(C)** Conducted with VOSviewer, this map presents keyword co-occurrence analysis, showing the network structure and clustering relationships between keywords, reflecting associations and distributions across different themes in the research field. **(D)** Generated using CiteSpace, this map depicts keyword burst detection, visually demonstrating the research popularity and evolution trends of keywords over time, emphasizing critical research directions that have gained attention in recent years. The size of the circles represents the number of articles associated with a term, while the proximity of related terms indicates their correlation based on co-occurrence frequency.

Using Bibliometrix to analyze the keywords of the article, you can obtain the current research hotspots. Select “KeyWords Plus” in Bibliometrix, there are a total of 5,339 keywords, of which the top ten are neuropathic pain, expression, activation, spinal-cord, glial activation, mechanical allodynia, peripheral-nerve injury, and rat model, nerve injury, neurons (Figures 4A,B). Use VOSviewer software to perform keyword co-occurrence analysis, select “All keywords,” and set the threshold of connection degree to 30, a total of 180 keywords meet the requirements, and obtain a cluster map of keyword co-occurrence, forming a total of 5 clusters of red, green, blue, yellow and purple (Figure 4C).

Using CiteSpace can obtain an explosion map of keywords, which can visually obtain research hotspots in recent years. Figure 4D showing the top 25 keywords for the strongest citation explosion between 2003 and 2022. Notably, keywords such as “satellite glia” (2018–2022), “oxidative stress” (2018–2022), and “neuroinflammation” (2020–2022) have received a lot of attention in recent years, suggesting that future research may be carried out in these areas.

## 4 Discussion

This study explores the research hotspots in the mechanisms of neuropathic pain involving glial cells, providing researchers and clinicians with an overview of potential future research directions in this field. A comprehensive search was conducted in the Web of Science Core Collection for literature published on this topic between 2003 and 2022. A total of 2,934 articles were retrieved and analyzed using bibliometric methods. Analysis of the annual publication trends for articles on glial cells and neuropathic pain reveals a growing interest in this research area. From 2003 to 2022, the number of published papers increased steadily, although the annual number of new publications remained below 250, indicating a modest growth rate. The highest number of publications occurred in 2022, with 231 papers. This upward trend reflects an increasing focus on this topic, gradually establishing it as a research hotspot. In terms of publications by countries and regions, 56 countries or regions contributed to this field. China published the highest number of articles. Regarding international collaboration, the United States ranked first in collaboration frequency with other countries. However, this ranking may be influenced by several factors: first, the reliance on the Web of Science database, which predominantly indexes English-language literature, potentially underestimates contributions from non-English-speaking countries such as Germany and Russia. Second, collaboration frequency was calculated based on co-authorship between countries, with multinational collaborations simplified into pairwise records, potentially undervaluing the overall contribution of multilateral efforts. Third, the influence of countries or institutions may vary dynamically over time, and cumulative rankings may obscure these temporal trends. Future research could address these limitations by incorporating multilingual literature and time-series analyses (25). Institutional analysis revealed that six of the top ten institutions by publication volume were in the United States. The University of California system ranked first with 130 publications, followed by the University of Colorado Boulder with 120 publications. Other leading institutions were located in China and France. The prominence of institutions like the University of California system and the University of Colorado Boulder in this field is attributed to several factors: long-standing expertise and resources in neuroscience, pain research, and basic medicine; interdisciplinary research platforms integrating biology, neuroscience, and clinical medicine; and robust international collaboration networks and research funding support, enabling cutting-edge studies. Analyzing the journals with the most publications revealed which journals were key contributors to this field and provided valuable insights for researchers seeking appropriate venues for their work. Pain emerged as the most prolific and most-cited journal in this field. Its prominence is due to its precise focus on pain mechanisms, diagnosis, and treatment; its rigorous peer-review process, ensuring high-quality and credible research; and its appeal to leading global research teams, making it the preferred platform for impactful publications. These factors collectively enhance the journal’s academic influence and citation frequency.

Identifying research hotspots is crucial for researchers to stay informed about current trends, a primary goal of bibliometric analysis. In the study of neuropathic pain and glial cells, this research highlights the intricate interplay between glial cells and neuropathic pain mechanisms. Glial cells, including microglia, astrocytes, oligodendrocytes, and satellite glial cells, play critical roles in the initiation and maintenance of neuropathic pain. Microglia, the resident macrophages of the spinal cord and brain, are rapidly activated by even minor pathological changes in the central nervous system. Peripheral nerve injury induces significant microglial proliferation and activation, accompanied by upregulation of markers such as IBA1 and CD11b in the spinal cord (26–29). Activated microglia release pro-inflammatory factors (e.g., IL-1β, TNF-*α*, and IL-6) and chemokines, leading to neuroinflammation and pain hypersensitivity (30–32). Activation also involves the upregulation of P2X4 and P2X7 receptors, which are closely associated with hyperalgesia (33). Astrocytes perform numerous essential functions, including neurotransmitter cycling, maintaining the blood–brain barrier, regulating extracellular ion concentrations, and modulating synaptic transmission. Nerve injury induces changes in astrocytes, impairing their ability to maintain stable extracellular potassium and glutamate levels, leading to neuronal hyperexcitability (34). The downregulation of potassium-chloride cotransporter 2 (KCC2), a protein essential for chloride ion transport, is associated with the generation of neuropathic pain. KCC2 functions as a potassium-dependent chloride ion exporter, relying on the concentration gradient of potassium ions inside and outside the neuron. Its activity requires a low extracellular potassium ion concentration, a condition maintained primarily by astrocytes. Astrocytes also secrete inflammatory factors and chemokines in response to nerve injury, exacerbating pain (35, 36). Oligodendrocyte-derived IL-33 contributes to hypersensitivity via MAP kinase and NF-κB signaling pathways (37). Satellite glial cells in sensory ganglia play an essential role in pain signaling by releasing inflammatory mediators and interacting with neurons, especially in peripheral nerve injury-induced pain sensitization (38, 39).

Citation analysis highlighted the academic influence of key articles in this field. Among the globally and locally most-cited articles, SCHOLZ J et al.’s paper The neuropathic pain triad: neurons, immune cells, and glia (published in Nature Neuroscience) was cited 1,275 times. This study introduced the “neuropathic pain triad,” emphasizing the interaction of neurons, immune cells, and glial cells in neuropathic pain. It underscored the central role of neuroinflammation and glial activation in pain maintenance and influenced future studies targeting glial cells for therapeutic strategies.

Keyword analysis revealed core research themes. High-frequency keywords included “spinal cord,” “glial cell activation,” “mechanical allodynia,” and “peripheral nerve injury.” Co-occurrence clustering identified five major themes. Recent research hotspots, as indicated by CiteSpace, include oxidative stress, neuroinflammation, and satellite glial cells. Oxidative stress is a core mechanism of neuropathic pain, with excessive reactive oxygen species (ROS) triggering glial activation and inflammatory mediator release (40). Neuroinflammation, characterized by glial activation and excessive pro-inflammatory cytokines like TNF-*α*, IL-1β, and IL-6, perpetuates pain (41, 42). Emerging therapeutic strategies include P2X4/P2X7 receptor antagonists (33), NADPH oxidase inhibitors (43), and IL-33 pathway blockers (44). These therapies target glial activation and inflammatory signaling pathways, offering hope for neuropathic pain management.

This study comprehensively explores research trends and hotspots in the field of neuropathic pain and glial cells through bibliometric analysis. Compared to traditional literature review methods, this research adopts a systematic search and a multi-tool approach, integrating CiteSpace, VOSviewer, and the bibliometrix R package. From data extraction and analysis to visualization, this methodology provides a more intuitive and systematic perspective. The Web of Science Core Collection database was selected for its high-quality content, extensive coverage, and authority in science, technology, engineering, and medicine (STEM) fields, ensuring a reliable source for this study. Additionally, Web of Science offers a wealth of bibliometric indicators, particularly for citation network analysis and research hotspot identification, supporting the discovery of key trends and academic collaboration networks in the field. However, this study has certain limitations. First, relying solely on Web of Science as the data source may limit coverage, as other databases such as PubMed and Scopus may contain high-quality studies not included here. Second, the study focuses exclusively on English-language literature. While this ensures consistency, it may overlook significant research published in non-English languages, such as German, French, Spanish, or Chinese, potentially introducing language bias. Third, the bibliometric analysis primarily relies on bibliographic information (e.g., titles, abstracts, and keywords) and does not delve into the full content of articles. As a result, key details, such as experimental methodologies or authors’ predictions about future research directions, may be missed. In terms of temporal scope, this study covers literature from 2003 to 2022, effectively capturing long-term trends and major developments in the field. However, emerging research dynamics beyond 2022 are not included. With the advancement of technology and research methodologies, new research hotspots may have emerged, such as the application of artificial intelligence in bibliometric analysis or the deepening of interdisciplinary research. These developments fall outside the scope of the current analysis and may limit its forward-looking perspective. To address these limitations, future research can improve in several directions. Expanding data sources to include databases like PubMed and Scopus could provide broader coverage and mitigate the limitations of relying on a single source. Incorporating non-English literature through translation tools or international collaborative studies could reduce language bias and capture more diverse academic perspectives. Additionally, using full-text semantic analysis techniques would enable a deeper exploration of content, including experimental details, author insights, and predictions about future trends. Finally, adopting a dynamic and regularly updated bibliometric analysis framework could track the latest advancements in the field, especially emerging trends after 2022, such as multi-database integration, multilingual studies, and enhanced interdisciplinary collaborations, thus improving the timeliness and foresight of the research. In conclusion, this study provides profound insights into research hotspots and trends in the field of neuropathic pain and glial cells, based on a systematic bibliometric analysis. Despite limitations such as reliance on a single data source, language bias, and temporal constraints, future studies can enhance comprehensiveness and depth through data integration, multilingual analysis, and full-text exploration. These improvements will offer more precise and dynamic support for the ongoing development of this research domain.

## 5 Conclusion

Our Bibliometric analysis should help researchers understand trends in neuralgia and glial cell research. A relatively small number of articles on this field are published each year, but they have matured in recent years. In short, our focus on neuralgia and glial cells as a field of research is gradually increasing, and more research will be done to improve the knowledge in this field in the future.

## Data Availability

The original contributions presented in the study are included in the article/supplementary material, further inquiries can be directed to the corresponding authors.

## Footnotes

^1^ https://www.bibliometrix.org
